# Clinical outcomes and learning curve of robot-assisted percutaneous cannulated screw fixation for femoral neck fractures

**DOI:** 10.3389/fsurg.2026.1980063

**Published:** 2026-10-26

**Authors:** Hu Gong, Yang Shu, Te Li, Jibin Chen, Fanghui Zhang

**Affiliations:** 1 Department of Orthopedics, The Sixth Hospital of Wuhan, Affiliated Hospital of Jianghan University, Wuhan, China; 2 Department of Orthopedics, Combat-Trauma Treatment Center, General Hospital of Central Theater Command, Wuhan, Hubei, China

**Keywords:** cannulated screw fixation, clinical outcomes, femoral neck fracture, learning curve, orthopaedic robotics

## Abstract

**Background:**

Robot-assisted cannulated-screw fixation may improve trajectory control and reduce fluoroscopic acquisitions during femoral neck fracture treatment; however, its comparative clinical benefits and early learning curve remain insufficiently characterized.

**Methods:**

This single-centre retrospective cohort study included 61 patients aged 18–65 years with acute Pauwels type II or III femoral neck fractures treated between January 2023 and January 2025. Twenty-one patients underwent robot-assisted fixation and 40 underwent conventional freehand fixation with three cannulated screws. Perioperative, radiographic, and 12-month clinical outcomes were compared. The learning curve was assessed using Spearman rank correlation, simple linear regression, and mean-centred cumulative sum (CUSUM) analysis.

**Results:**

Baseline characteristics were comparable between groups. Compared with conventional freehand fixation, robot-assisted fixation was associated with shorter total operative time., fewer fluoroscopic acquisitions.., and lower intraoperative blood loss… Robot-assisted fixation was associated with more favourable screw-placement parameters and less femoral-neck shortening. Postoperative day-1 pain scores assessed using VAS were lower in the robot-assisted group. HHS values were higher at 1 month and final follow-up. Fracture-healing time and neck–shaft angle did not differ significantly between groups. Only one postoperative complication occurred during follow-up, consisting of screw back-out in one patient in the freehand group; therefore, comparative safety conclusions were not supported. Within the robot-assisted cohort, case number was inversely associated with total operative time, puncture workflow time, and fluoroscopy use. The mean-centred CUSUM trajectories reached sample-specific maxima at cases 7, 6, and 6, respectively, before showing overall downward trajectories.

**Conclusion:**

Robot-assisted percutaneous cannulated-screw fixation was associated with greater procedural efficiency and more favourable screw-placement parameters than conventional freehand fixation. Statistically significant differences were observed in postoperative pain, HHS, and femoral-neck shortening; however, the clinical importance of these differences remains uncertain. Operative performance improved across the observed sequence of robot-assisted cases, although no validated proficiency threshold could be established from this cohort.

## 1 Introduction

Femoral neck fractures are common hip injuries and represent a substantial clinical burden, particularly among older adults (1, 2). Their intracapsular location and vulnerable blood supply increase the risk of complications such as nonunion and osteonecrosis of the femoral head, making preservation of the native hip challenging (3, 4). In older adults with displaced femoral neck fractures, a recent propensity score-matched study found that total hip arthroplasty was associated with better postoperative hip function, but longer operative time and greater blood loss, than hemiarthroplasty (5). In younger patients and selected patients requiring joint preservation, percutaneous fixation with cannulated screws remains widely used because it minimizes soft-tissue disruption and provides effective fracture stabilization.

Conventional freehand fixation relies heavily on the surgeon's interpretation of two-dimensional fluoroscopic images and repeated adjustment of guidewire trajectories. This operator-dependent workflow may increase fluoroscopic requirements and contribute to variation in screw positioning and final construct configuration (6, 7). Accurate placement of multiple parallel screws within the femoral neck is technically demanding, particularly in fractures with complex geometry or limited fluoroscopic visualization. Orthopaedic robotic systems integrate image-based planning, intraoperative navigation, and mechanically guided execution to assist implant placement (8–10). In femoral neck fixation, robotic assistance may facilitate guidewire and screw placement by improving trajectory control and reducing repeated fluoroscopic confirmation. However, these potential advantages require additional procedural steps, including tracker placement, image registration, and trajectory planning. The influence of these technical requirements on procedural performance and the acquisition of surgical proficiency remains insufficiently characterized.

Previous studies of robotic-assisted fracture fixation have mainly focused on perioperative efficiency and implant-placement accuracy, whereas changes in operative performance across consecutive cases have received less attention. Understanding the learning process associated with robotic-assisted fixation is important because the introduction of new technology may initially increase procedural complexity. However, the pattern and timing of performance changes during robot-assisted fixation of femoral neck fractures remain unclear.

In this retrospective cohort study, we compared perioperative, radiographic, and clinical outcomes between robot-assisted and conventional freehand percutaneous fixation with three cannulated screws in patients with femoral neck fractures. We assessed perioperative efficiency, radiographic parameters related to alignment and screw placement, postoperative pain, hip function, and complications. In addition, we evaluated changes in operative performance across consecutive robot-assisted cases using correlation analysis, regression analysis, and cumulative sum (CUSUM) analysis to characterize the early learning curve associated with robot-assisted fixation.

## 2 Methods

### 2.1 Study design and participants

This retrospective cohort study included patients with femoral neck fractures who underwent surgical treatment at the Sixth Hospital of Wuhan between January 2023 and January 2025. Both robot-assisted and conventional freehand fixation cases were collected during the same study period following the introduction of the TiRobot system. Patients were classified according to the fixation technique received: robot-assisted percutaneous cannulated screw fixation or conventional freehand percutaneous cannulated screw fixation. A total of 61 patients were included, comprising 21 patients in the robot-assisted group and 40 patients in the freehand group.

The TiRobot system was introduced at our institution in 2023. After its introduction, patients with femoral neck fractures requiring percutaneous cannulated screw fixation were informed of both robot-assisted and conventional freehand fixation options. Treatment allocation was not randomized and was determined through shared decision-making between the surgeon and patient, considering patient preference, acceptance of robotic assistance, and the additional financial costs associated with robotic procedures. Treatment selection was not influenced by fracture complexity or surgeon preference.

The study protocol was approved by the Ethics Committee of the Sixth Hospital of Wuhan (approval no.WHSHIRB-K-2026005). Demographic and clinical data were obtained from medical records and the institutional imaging system. Baseline variables collected included age, sex, body mass index (BMI), fracture laterality, Garden classification, Pauwels classification, and time from injury to surgery.

### 2.2 Eligibility criteria

Patients were eligible for inclusion if they met the following criteria: age 18–65 years; acute femoral neck fracture classified as both Garden type II or III and Pauwels type II or III; surgery performed within 2 weeks after injury; successful closed reduction followed by percutaneous fixation with three cannulated screws; and availability of complete clinical, radiographic, and follow-up data.

Garden type I fractures were excluded because valgus-impacted or incomplete fractures represent a distinct biomechanical and clinical subgroup and may not require the same reduction and fixation strategy as Garden type II or III fractures. Garden type IV fractures were excluded to maintain a relatively homogeneous cohort of fractures suitable for closed reduction and three-screw percutaneous fixation.

### 2.3 Surgical procedures

All 21 robot-assisted procedures were performed by the same primary surgeon with the same trauma orthopaedic surgical team throughout the study period. The primary surgeon had extensive prior experience with conventional percutaneous cannulated screw fixation and completed manufacturer-provided TiRobot training before performing robot-assisted procedures. The training included system operation, preoperative planning, registration, and navigation workflows. Closed reduction was achieved using sequential abduction and external rotation followed by adduction and internal rotation of the affected limb. Reduction was evaluated using anteroposterior (AP) and lateral fluoroscopy, and percutaneous Kirschner-wire manipulation was performed when additional adjustment was required.

The robot-assisted group underwent fixation using the third-generation TiRobot orthopaedic robotic system. The system consists of four main components: a robotic-arm manipulation subsystem, an optical tracking and positioning subsystem, a surgical planning subsystem, and an intraoperative real-time navigation subsystem.

#### 2.3.1 Robot-assisted fixation

A tracking device was fixed securely to the anterior superior iliac spine, and a robotic-arm tracker was positioned within the operative field. After satisfactory fracture reduction had been confirmed fluoroscopically, anteroposterior and lateral images of the femoral neck were acquired using a C-arm and transferred to the robotic workstation. The trajectories of the three guidewires were planned using the reconstructed images.

After the robotic arm was aligned with the planned trajectories, three skin incisions measuring approximately 0.5–1.0 cm were created. Guidewires were inserted percutaneously through the robotic-arm sleeve. Fluoroscopy was used to confirm that the guidewire tips were positioned approximately 5 mm beneath the subchondral bone. Screw length was subsequently measured, and three appropriately sized cannulated screws were inserted along the planned trajectories, approximately parallel to the femoral neck axis. Compression was applied when required. Final fluoroscopic images were obtained to confirm implant position and fixation status. The incisions were then closed without placement of a drainage tube (Figure 1).

**Figure 1:**
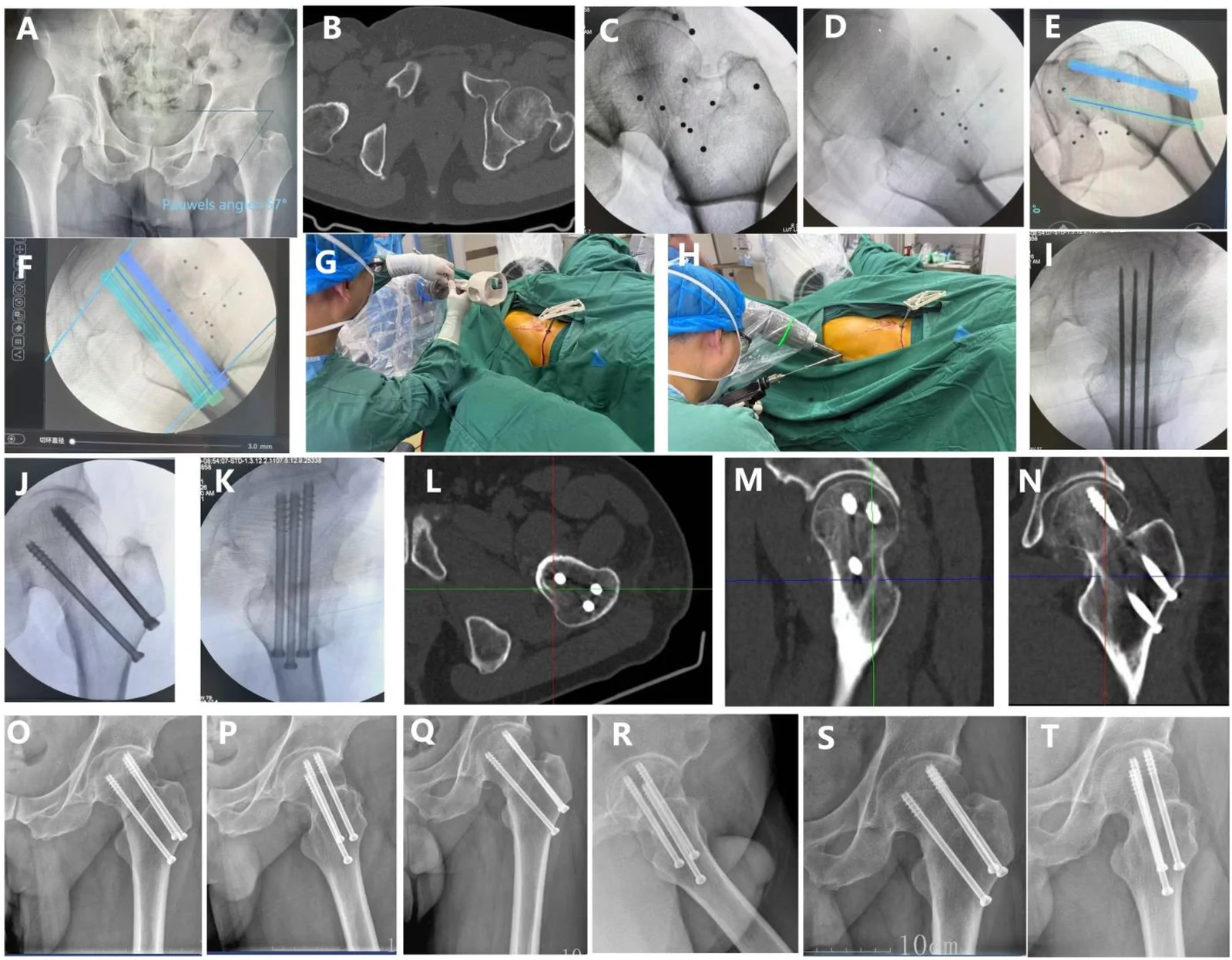
Representative imaging, robot-assisted surgical workflow and postoperative radiographic follow-up. **(A,B)** Preoperative anteroposterior radiograph and computed tomography image showing the femoral neck fracture. The Pauwels angle was indicated on the preoperative radiograph to demonstrate the fracture inclination pattern. **(C,D)** Intraoperative fluoroscopic images confirming that all radiopaque fiducial markers were included within the field of view for image registration. **(E,F)** Robotic localization and trajectory planning based on intraoperative fluoroscopic imaging. **(G-I)** Placement of the tracking device and robot-guided insertion of three guidewires. **(J,K)** Intraoperative fluoroscopic images demonstrating satisfactory fracture reduction and appropriate screw positioning following cannulated screw fixation. **(L-N)** Immediate postoperative computed tomography images confirming satisfactory reduction and screw placement in the axial, coronal and sagittal planes. **(O,P)** Immediate postoperative anteroposterior and lateral radiographs showing maintained reduction and appropriate screw position. **(Q,R)** Anteroposterior and lateral radiographs obtained 1 month after surgery, demonstrating maintained fracture alignment and stable screw position. **(S,T)** Anteroposterior and lateral radiographs obtained 3 months after surgery, showing maintained reduction and stable internal fixation.

#### 2.3.2 Conventional freehand fixation

After fluoroscopic confirmation of fracture reduction, the approximate entry points were determined using anteroposterior and lateral C-arm images. A skin incision of approximately 5 cm was made 3–4 cm distal to the greater trochanter. Three guidewires were manually inserted from the lateral femoral cortex toward the femoral neck under repeated fluoroscopic guidance. After satisfactory guidewire positioning was confirmed, screw lengths were measured and three cannulated lag screws were inserted. The incision was extended when necessary to facilitate guidewire manipulation and screw insertion. Final fluoroscopy was performed to confirm implant position and fixation. In the robot-assisted group, fixation was performed through three percutaneous incisions of approximately 0.5–1.0 cm with robotic trajectory guidance and intraoperative navigation. In the freehand group, fixation was performed using the conventional technique adopted at our institution, involving an approximately 5-cm lateral incision to facilitate fluoroscopic positioning, guidewire manipulation, and screw insertion.

### 2.4 Postoperative management and follow-up

Both groups received perioperative antibiotic prophylaxis. Anteroposterior (AP) and lateral radiographs of the affected hip were obtained within 48 h after surgery. After anaesthetic recovery, patients received intermittent pneumatic compression and were instructed to perform regular ankle-pump exercises. Limb swelling was assessed during routine ward rounds, and duplex ultrasonography was performed when clinically indicated to screen for lower-extremity deep-vein thrombosis.

Sutures were removed approximately 2 weeks after surgery. Patients underwent scheduled outpatient clinical and radiographic follow-up, and progressive weight bearing was allowed according to radiographic evidence of fracture healing. Clinical and functional outcomes were assessed throughout the 12-month follow-up period.

### 2.5 Outcome measures

Perioperative outcomes included total operative time, intraoperative blood loss, puncture workflow time, and the number of intraoperative fluoroscopic acquisitions. Total operative time was defined as the interval from the initial skin incision to completion of wound closure. Puncture workflow time was defined as the interval from initiation of guidewire localization in the freehand group or trajectory planning in the robot-assisted group to completion of placement of all three guidewires. In the robot-assisted group, robot setup, tracker placement, image acquisition, and registration performed before trajectory planning were not included in the puncture workflow time. A single fluoroscopic acquisition was defined as one captured anteroposterior or lateral fluoroscopic image. Intraoperative blood loss was estimated based on the volume collected in the suction system after subtraction of irrigation fluid, together with the estimated blood volume retained in used surgical gauze and sponges, as documented in the operative and anaesthesia records.

Radiographic outcomes included anteroposterior and lateral Garden alignment indices, neck–shaft angle, cannulated-screw parallelism angle, screw-dispersion angle, and femoral-neck shortening. The Garden alignment indices, neck–shaft angle, screw-parallelism angle, and screw-dispersion angle were measured on anteroposterior and lateral radiographs obtained within 48 h after surgery. Femoral-neck shortening was assessed on radiographs obtained at the final 12-month follow-up by comparison with the contralateral uninjured hip as an internal reference.

Radiographic fracture healing was defined as blurring or disappearance of the fracture line, or bridging of the fracture line by continuous callus formation, with no evidence of deformity or refracture on serial radiographs obtained over at least two consecutive weeks.

Postoperative clinical outcomes included fracture-healing time, postoperative complications, pain intensity assessed using the Visual Analogue Scale (VAS) on postoperative day 1 (11), and hip function assessed using the Harris Hip Score (HHS) at 1 month and final follow-up (12). Postoperative complications were recorded throughout the 12-month follow-up period based on routine clinical assessments and radiographic examinations. Complications of interest included osteonecrosis of the femoral head, nonunion, fixation-related failure, reoperation, and conversion to hip arthroplasty.

All radiographic measurements were independently performed by two orthopaedic surgeons trained in the measurement protocol. Before assessment, all radiographs were anonymized by removing patient names, medical-record identifiers, and treatment-group information. Both assessors independently evaluated all 61 cases. To assess intraobserver repeatability, one assessor repeated the measurements after a 2-week interval.

### 2.6 Learning curve analysis

Puncture workflow time was selected as the primary indicator of procedural learning because it reflects the core technical workflow associated with guidewire placement. Cases in the robot-assisted and freehand groups were indexed separately according to the chronological sequence of surgery within each group. Spearman rank correlation analysis was initially performed to evaluate the association between case number and procedural performance metrics, and the resulting P values were adjusted for multiple comparisons using the Holm procedure.

Simple linear regression analysis was subsequently performed to quantify the relationship between case number and total operative time, puncture workflow time, and the number of fluoroscopic acquisitions. Regression coefficients and corresponding 95% confidence intervals were calculated.

The learning curve was further explored using cumulative sum (CUSUM) analysis. CUSUM values were calculated based on consecutive cases performed by the same primary surgeon in each group according to the following formula:

$$\mathrm{CUSU}M_{n}=\overset{n}{\underset{i\;=\;1}{\sum }}\;(X_{i}-\bar{X})$$

where ∖(x_i∖) represents the observed value for the i-th case, ∖(∖bar{x}∖) represents the mean value of the corresponding outcome within the group, and n represents the chronological case number. An upward CUSUM trajectory indicates that consecutive observations are predominantly above the group mean, whereas a downward trajectory indicates that observations are predominantly below the group mean. The CUSUM analysis was performed as a descriptive exploratory assessment of chronological changes in procedural performance. No predefined acceptable or unacceptable performance limits were established; therefore, CUSUM maxima were not interpreted as validated proficiency thresholds or formal change points.

In addition, the robot-assisted cohort was divided into three consecutive stages of equal size according to surgical sequence. Changes in total operative time, puncture workflow time, and the number of fluoroscopic acquisitions across these stages were evaluated to describe temporal trends in procedural performance. To improve transparency regarding potential temporal and case-mix effects, the 21 robot-assisted cases are presented individually in chronological order in Supplementary Table S1, including major patient and fracture characteristics and the three procedural performance outcomes.

### 2.7 Statistical analysis

Statistical analyses were performed using IBM SPSS Statistics version 26.0. Continuous variables were assessed for normality using the Shapiro–Wilk test and visual inspection of data distributions. Normally distributed continuous variables were expressed as mean ± standard deviation (SD), whereas non-normally distributed variables were expressed as median and interquartile range (IQR).Between-group comparisons of normally distributed continuous variables were performed using independent-samples t-tests. Homogeneity of variance was assessed using Levene's test, and Welch's *t*-test was applied when the assumption of equal variances was violated. Non-normally distributed continuous variables were compared using the Mann–Whitney U test.

Categorical variables were presented as counts and percentages. Binary categorical variables were compared using Pearson's chi-square test or Fisher's exact test when expected cell counts were small. Garden and Pauwels classifications were treated as categorical variables; because only type II and type III fractures were included in this study, distributions between groups were compared using the chi-square test.All statistical tests were two-sided, and *P* < 0.05 was considered statistically significant. Statistical methods specific to the learning-curve analysis are described separately in Section 2.6. Total operative time was predefined as the primary endpoint, whereas all other outcomes were considered secondary endpoints.

## 3 Results

### 3.1 Baseline characteristics

Sixty-one patients were included, comprising 21 patients in the robot-assisted group and 40 patients in the conventional freehand group. All patients underwent the planned surgical procedure and completed the 12-month follow-up. Baseline demographic and fracture-related characteristics are summarized in Table 1. No statistically significant differences were observed between groups in age (52.29 ± 2.83 versus 52.70 ± 2.72 years; t = −0.557, *P* = 0.579), body mass index (BMI) (22.92 ± 1.55 versus 22.87 ± 1.30 kg/m^2^; t = 0.121, *P* = 0.904), sex distribution (male/female, 9/12 versus 21/19; *χ*^2^ = 0.512, *P* = 0.474), fracture laterality (left/right, 8/13 versus 23/17; *χ*^2^ = 2.075, *P* = 0.150), Garden classification (type II/III, 14/7 versus 27/13; *χ*^2^ = 0.004, *P* = 0.947), or Pauwels classification (type II/III, 12/9 versus 25/15; *χ*^2^ = 0.166, *P* = 0.684). The interval from injury to surgery was also similar between groups (4.00 [3.00–5.00] versus 4.00 [3.00–4.00] days; Z = 0.95, *P* = 0.341).

**Table 1:** Baseline characteristics of the study cohort.

Characteristic	Robot-assisted (*n* = 21)	Freehand (*n* = 40)	Test statistic	P value
Age, years	52.29 ± 2.83	52.70 ± 2.72	t = −0.557	0.579
Body mass index, kg/m^2^	22.92 ± 1.55	22.87 ± 1.30	t = 0.121	0.904
Sex, male/female	9/12	21/19	*χ*^2^ = 0.512	0.474
Fracture laterality, left/right	8/13	23/17	χ^2^ = 2.075	0.150
Garden classification, II/III	14/7	27/13	χ^2^ = 0.004	0.947
Pauwels classification, II/III	12/9	25/15	χ^2^ = 0.166	0.684
Time from injury to surgery, days	4.00 (3.00–5.00)	4.00 (3.00–4.00)	Z = 0.952	0.341

Data are mean ± s.d., median (interquartile range) or counts. P values were calculated using two-sided independent-samples t-tests, χ^2^ tests or Mann–Whitney U tests, as appropriate.

### 3.2 Robot-assisted fixation was associated with shorter operative time, lower blood loss and fewer fluoroscopic acquisitions

Robot-assisted fixation was associated with shorter total operative time compared with freehand fixation (65.05 ± 4.19 versus 77.93 ± 5.62 min; t = −9.223, *P* < 0.001; Table 2). Puncture workflow time was also shorter in the robot-assisted group than in the freehand group (5.00 [5.00–7.00] versus 12.00 [11.00–12.25] min; Z = −6.283, *P* < 0.001), and fewer fluoroscopic acquisitions were observed (12.00 [10.00–15.00] versus 17.50 [16.00–20.00]; Z = −5.552, *P* < 0.001). Intraoperative blood loss was lower in the robot-assisted group than in the freehand group (23.00 [22.00–28.00] versus 34.50 [29.50–38.00] mL; Z = −4.895, *P* < 0.001).

**Table 2:** Perioperative and radiographic outcomes.

Characteristic	Robot-assisted (*n* = 21)	Freehand (*n* = 40)	Test statistic	P value
Total operative time, min	65.05 ± 4.19	77.93 ± 5.62	t = −9.223	**<0** **.** **001**
Intraoperative blood loss, mL	23.00 (22.00–28.00)	34.50 (29.50–38.00)	Z = −4.895	**<0**.**001**
Puncture workflow time, min	5.00 (5.00–7.00)	12.00 (11.00–12.25)	Z = −6.283	**<0**.**001**
Fluoroscopy count	12.00 (10.00–15.00)	17.50 (16.00–20.00)	Z = −5.552	**<0**.**001**
Garden alignment index, AP,°	161.00 (159.00–164.00)	157.50 (155.00–160.25)	Z = 3.571	**<0**.**001**
Garden alignment index, lateral,°	179.00 (178.00–180.00)	177.50 (176.00–179.00)	Z = 2.643	**0**.**008**
Neck–shaft angle,°	132.67 ± 2.24	132.38 ± 2.00	t = 0.520	0.605
Screw-parallelism angle,°	1.90 (1.70–2.10)	2.15 (2.00–2.30)	Z = −2.646	**0**.**008**
Screw-dispersion angle,°	73.80 (73.20–74.10)	70.00 (69.30–70.62)	Z = 6.211	**<0**.**001**
Femoral-neck shortening, mm	4.30 (4.20–4.30)	5.25 (4.90–5.53)	Z = −6.379	**<0**.**001**

Data are mean ± s.d. or median (interquartile range). AP, anteroposterior. P values were calculated using two-sided independent-samples t-tests or Mann–Whitney U tests, as appropriate.

Bold values indicate statistically significant differences between groups (*P* < 0.05).

### 3.3 Radiographic and screw-placement parameters after fixation

Postoperative radiographic assessment showed higher anteroposterior (AP) Garden alignment indices in the robot-assisted group compared with the freehand group (161.00° [159.00–164.00°] versus 157.50° [155.00–160.25°]; Z = 3.57, *P* < 0.001), as well as higher lateral Garden alignment indices (179.00° [178.00–180.00°] versus 177.50° [176.00–179.00°]; Z = 2.64, *P* = 0.008). Robot-assisted fixation was associated with a smaller screw-parallelism angle (1.90° [1.70–2.10°] versus 2.15° [2.00–2.30°]; Z = −2.65, *P* = 0.008), a larger screw-dispersion angle (73.80° [73.20–74.10°] versus 70.00° [69.30–70.63°]; Z = 6.21, *P* < 0.001), and reduced femoral-neck shortening (4.30 [4.20–4.30] versus 5.25 [4.90–5.53] mm; Z = −6.38, *P* < 0.001). The neck–shaft angle did not differ significantly between groups (132.67 ± 2.24° versus 132.38 ± 2.00°; t = 0.520, *P* = 0.605).

### 3.4 Postoperative clinical outcomes and complications

Fracture-healing time was similar between the robot-assisted and freehand groups (18.00 [18.00–18.00] versus 18.00 [18.00–18.00] weeks; Z = −0.92, *P* = 0.359; Table 3). During the 12-month follow-up period, no postoperative complications were recorded in the robot-assisted group. One patient in the freehand group experienced screw back-out (0/21 [0.0%] versus 1/40 [2.5%]; Fisher's exact test, *P* = 1.000). No cases of osteonecrosis of the femoral head, nonunion, other fixation-related failure, or conversion to hip arthroplasty were observed during the follow-up period. Given that only one complication occurred in the entire cohort, meaningful conclusions regarding comparative safety could not be drawn. Postoperative day-1 pain scores were lower in the robot-assisted group than in the freehand group (VAS score, 3.00 [2.00–3.00] versus 4.00 [3.00–5.00]; Z = −3.67, *P* < 0.001). HHS values were higher in the robot-assisted group at 1 month (85.00 [84.00–86.00] versus 83.00 [82.00–84.00]; Z = 4.35, *P* < 0.001) and at the final follow-up (88.62 ± 2.16 versus 86.78 ± 1.94; t = 3.393, *P* = 0.001).

**Table 3:** Postoperative clinical outcomes.

Characteristic	Robot-assisted (*n* = 21)	Freehand (*n* = 40)	Test statistic	P value
Any postoperative complication, n/N (%)	0/21 (0.0%)	1/40 (2.5%)	—	1.000
Fracture-healing time, weeks	18.00 (18.00–18.00)	18.00 (18.00–18.00)	Z = −0.918	0.359
VAS score, postoperative day 1	3.00 (2.00–3.00)	4.00 (3.00–5.00)	Z = −3.673	**<0** **.** **001**
HHS, 1 month	85.00 (84.00–86.00)	83.00 (82.00–84.00)	Z = 4.351	**<0**.**001**
HHS, final follow-up	88.62 ± 2.16	86.78 ± 1.94	t = 3.393	**0**.**001**

Data are presented as mean ± s.d., median (interquartile range) or n/N (%), as appropriate. P values were calculated using two-sided Mann–Whitney U tests, independent-samples t-tests or Fisher's exact test, as appropriate. The only postoperative complication was screw back-out in one patient in the freehand group during the 12-month follow-up.

VAS, visual analogue scale; HHS, Harris hip score.

Bold values indicate statistically significant differences between groups (*P* < 0.05).

### 3.5 Operative performance changes across consecutive robot-assisted cases

Within the robot-assisted cohort, increasing case number was inversely associated with total operative time, puncture workflow time, and the number of fluoroscopic acquisitions. Spearman rank correlation coefficients were −0.728, −0.612, and −0.558, respectively, and all associations remained statistically significant after Holm correction (adjusted *P* = 0.0005, 0.0064, and 0.0086, respectively). Simple linear regression analysis showed consistent findings, with each additional robot-assisted case associated with decreases of 0.514 min in total operative time (95% CI, 0.304–0.724), 0.169 min in puncture workflow time (95% CI, 0.079–0.259), and 0.252 fluoroscopic acquisitions (95% CI, 0.101–0.403). The corresponding mean-centred CUSUM trajectories reached sample-specific maxima at cases 7, 6, and 6 for total operative time, puncture workflow time, and fluoroscopic acquisitions, respectively, followed by overall downward trajectories (Figure 2). These maxima were considered descriptive features of the observed case sequence and were not interpreted as validated proficiency thresholds or formal change points. Compared with the first tertile of robot-assisted cases, the final tertile showed decreases of 11.2% in total operative time, 36.0% in puncture workflow time, and 23.1% in the number of fluoroscopic acquisitions. In contrast, none of these outcomes showed a significant association with case number in the freehand group (Spearman *ρ* = 0.289, 0.003, and 0.032; all Holm-adjusted *P* > 0.05). The corresponding CUSUM trajectories fluctuated without a sustained downward trend after the observed maxima.

**Figure 2:**
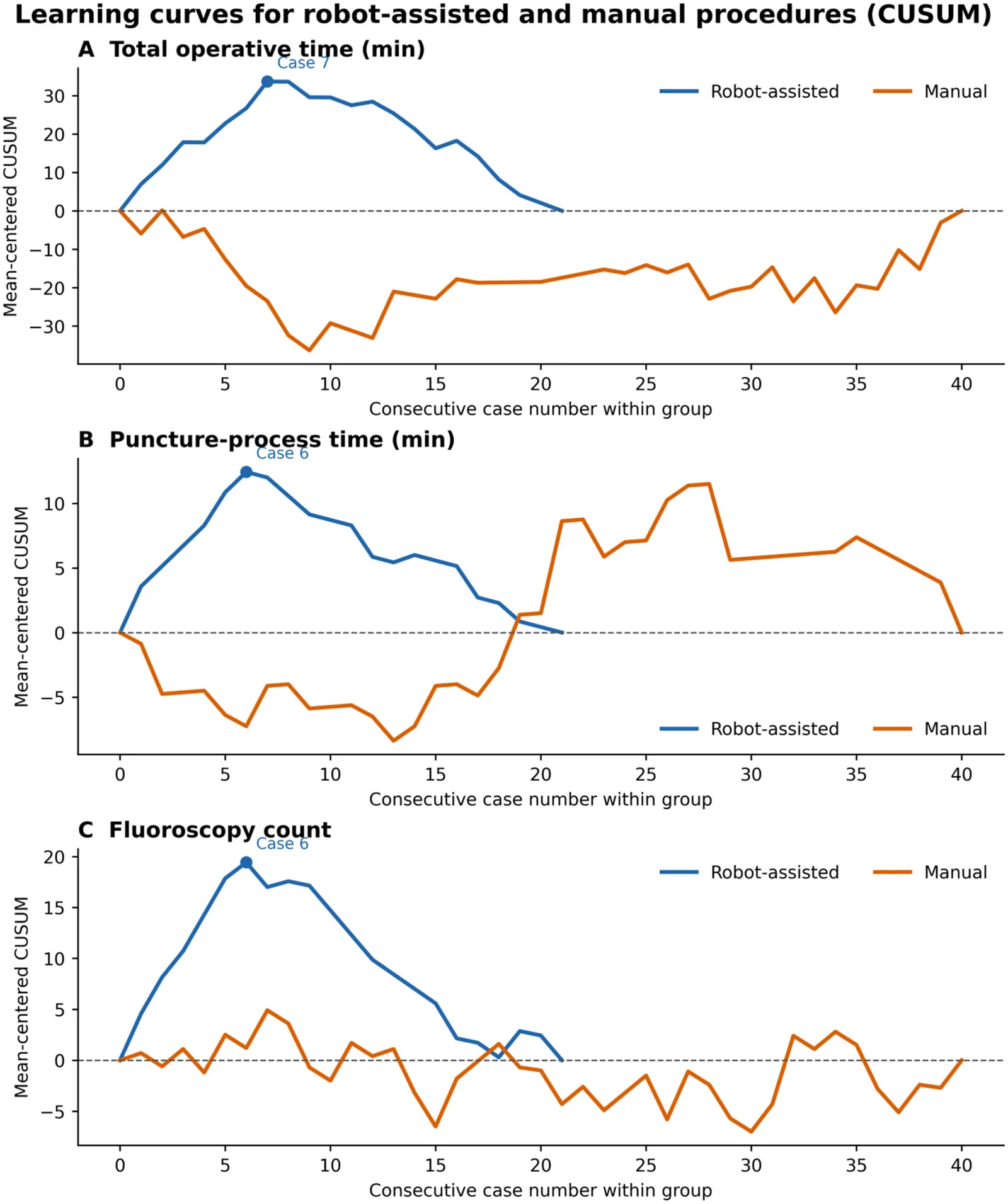
CUSUM learning curves for robot-assisted and freehand fixation. Mean-centred cumulative sum plots for **(A)** total operative time, **(B)** puncture workflow time and **(C)** fluoroscopy count. Blue and orange lines denote robot-assisted and freehand procedures, respectively. Labels indicate the maxima of the robot-assisted curves: case 7 for total operative time and case 6 for both puncture workflow time and fluoroscopy count. The subsequent downward trajectories in the robot-assisted group are consistent with improving procedural efficiency across consecutive cases, whereas the freehand curves show irregular fluctuations without a sustained post-peak decline. Dashed horizontal lines indicate zero. These maxima represent sample-specific descriptive features of the mean-centred CUSUM trajectories and should not be interpreted as validated proficiency thresholds or formal change points.

## 4 Discussion

This retrospective cohort study suggests that robot-assisted percutaneous fixation with three cannulated screws may improve procedural efficiency and enhance the consistency of implant placement in selected patients with femoral neck fractures. Compared with conventional freehand fixation, robot-assisted fixation was associated with shorter total operative time and puncture workflow time, lower intraoperative blood loss, and fewer fluoroscopic acquisitions. The robot-assisted group also demonstrated more favourable radiographic alignment parameters and screw-placement characteristics, including Garden alignment indices, screw parallelism, screw dispersion, and reduced femoral-neck shortening. These technical differences were accompanied by lower postoperative day-1 pain scores and higher HHS values at 1 month and final follow-up, whereas fracture-healing time, neck–shaft angle, and complication frequency were similar between groups. Taken together, these findings suggest that the potential value of robotic assistance in this setting may lie primarily in facilitating more consistent guidewire and screw placement and reducing intraoperative adjustments rather than accelerating biological fracture healing.

The reductions in operative time and fluoroscopic acquisitions may be clinically meaningful from a workflow perspective because conventional freehand cannulated-screw fixation relies on repeated interpretation of two-dimensional fluoroscopic images and adjustment of guidewire trajectories. This workflow is influenced by surgeon experience, patient positioning, fracture morphology, and fluoroscopic image quality (13, 14). By integrating image-based planning with mechanical guidance, the robotic system may facilitate more consistent execution of planned trajectories and reduce repeated guidewire adjustments and fluoroscopic confirmation (15, 16). In the present cohort, robot-assisted fixation was associated with a 12.88-min reduction in total operative time, a 6.45-min reduction in puncture workflow time, and 5.87 fewer fluoroscopic acquisitions per procedure. These findings are consistent with previous comparative studies and meta-analyses reporting reduced fluoroscopy use, blood loss, and operative burden with robot-assisted fixation (17–20). However, fluoroscopic acquisition number should be interpreted only as a surrogate measure of radiation exposure, as reductions in absorbed dose depend on imaging protocols, equipment characteristics, and the positioning of patients and operating personnel. In addition, robotic setup, tracker fixation, and image registration introduce additional procedural steps; whether these steps are offset by more efficient guidewire placement is likely to depend on team familiarity and workflow adaptation (21).

The radiographic findings provide a potential mechanical explanation for the observed differences in early postoperative outcomes, although causal relationships cannot be established from this retrospective comparison. Reduction quality and screw configuration are important determinants of construct stability in femoral neck fixation (22, 23) A Garden alignment index approaching 160° on the anteroposterior view and 180° on the lateral view is generally considered indicative of satisfactory alignment, while three screws are ideally positioned in a parallel configuration with broad distribution within the femoral neck. The higher Garden alignment indices, smaller screw-parallelism angle, and larger screw-dispersion angle observed in the robot-assisted group suggest that robotic assistance may have facilitated more consistent execution of planned guidewire trajectories and screw placement rather than simply reducing operative duration. The median between-group difference in femoral-neck shortening was approximately 0.95 mm. Although statistically significant, the clinical relevance of this magnitude of difference remains uncertain. A validated minimal clinically important difference for femoral-neck shortening has not been established. Previous studies have commonly used approximately 5 mm as a threshold for defining shortening and >10 mm as substantial shortening. Furthermore, because formal interobserver and intraobserver reliability analyses were not performed in the present study, measurement variability should be considered when interpreting this relatively small absolute difference. The absence of a between-group difference in neck–shaft angle further suggests that any potential benefit of robotic assistance was primarily related to guidewire and screw placement after satisfactory closed reduction had already been achieved.

Robot-assisted fixation was also associated with lower postoperative day-1 pain scores and higher HHS values during follow-up. Reduced repeated guidewire manipulation, less soft-tissue disruption, and more consistent screw placement may contribute to earlier pain relief and functional recovery; however, these potential mechanisms cannot be confirmed in this retrospective study. The persistence of a modest difference in HHS values at final follow-up is noteworthy, but its clinical relevance cannot be determined from statistical significance alone because a prespecified minimal clinically important difference was not assessed.

Fracture-healing time did not differ significantly between groups in the present study. In older patients with osteoporotic intertrochanteric fractures, Chen et al. reported that adding alfacalcidol to proximal femoral nail antirotation fixation was associated with higher bone mineral density at six months and shorter fracture-healing time (24). Although the fracture type and patient population differ from those in our study, these findings suggest that bone health and adjunctive medical treatment warrant consideration alongside fixation technique when interpreting recovery.

No postoperative complications occurred in the 21 robot-assisted cases, whereas one screw back-out event occurred among the 40 freehand cases during the 12-month follow-up period. Given the low event frequency and Fisher's exact test result (*P* = 1.000), these findings are insufficient to support conclusions regarding differences in safety outcomes between groups. Larger studies with longer follow-up are required to evaluate uncommon but clinically important outcomes, including osteonecrosis, nonunion, and fixation failure.

The learning-curve analysis further characterized temporal changes in robot-assisted procedural performance. Total operative time, puncture workflow time, and fluoroscopy use decreased with increasing case number, and these associations remained statistically significant after Holm correction. The CUSUM trajectories reached observed maxima at cases 7, 6, and 6, respectively, followed by an overall downward trend. The consistent direction of change across three related but distinct performance measures suggests that the observed pattern may reflect progressive adaptation to the robotic workflow rather than variation in a single outcome measure alone. However, given the limited number of robot-assisted cases and the retrospective design, the influence of temporal factors, case selection, and workflow changes cannot be excluded. The largest relative reduction was observed in puncture workflow time, suggesting that early performance changes were concentrated in steps closely related to robotic assistance, including tracker positioning, trajectory planning, robotic-arm alignment, and guidewire insertion. The concurrent reduction in fluoroscopic acquisitions may indicate reduced reliance on repeated image confirmation as familiarity with the workflow increased.

These CUSUM peaks should not be interpreted as validated proficiency thresholds. The analysis was descriptive and based on mean-centred cumulative deviations without prospectively defined acceptable or unacceptable performance limits. Therefore, the observed maxima around cases 6–7 should be regarded as sample-specific features of the observed case sequence rather than evidence of a discrete learning transition or the point at which proficiency was achieved. The absence of a comparable trend in the freehand group also does not establish a causal difference in learning trajectories between the two techniques. Freehand fixation may have already reached a mature stage of procedural experience before the observation period, and differences in case sequence, fracture complexity, or team composition may have influenced performance trends in either group. Future studies should preserve true chronological case order, incorporate relevant surgeon- and case-level covariates, and consider risk-adjusted CUSUM approaches with prespecified performance standards. Segmented regression and external validation may further clarify whether the observed changes represent a sustained transition toward a stable performance plateau.

This study has several limitations. First, the retrospective, single-centre design and unequal group sizes introduce potential selection bias, temporal bias, and residual confounding. Treatment allocation was not randomized, and the absence of significant baseline differences does not exclude unmeasured differences in fracture complexity, bone quality, or operative difficulty between groups. Second, the robot-assisted cohort included only 21 cases, limiting statistical precision and making the observed learning trajectory susceptible to individual-case variation. Although the freehand group did not demonstrate a sustained decline comparable to that observed in the robot-assisted group, an early change in the CUSUM trajectory for total operative time was observed around case 9. This suggests that repeated performance of the procedure may also improve operative efficiency through general procedural experience. Therefore, the observed robot-assisted learning pattern should be interpreted cautiously, as part of the improvement may reflect cumulative surgical experience, workflow adaptation, or team-level familiarity rather than robot-specific learning alone. Third, although all robot-assisted procedures were performed by the same primary surgeon and surgical team, the small learning-curve cohort limits the ability to separate robot-specific adaptation from other time-dependent factors, including case-mix variation and changes in workflow. Future studies with larger cohorts and risk-adjusted learning-curve analyses are required to further clarify these effects. Fourth, the statistical analysis was primarily based on unadjusted between-group comparisons and did not incorporate multivariable adjustment for potential confounders or longitudinal modelling of repeated functional outcomes. Fifth, the 12-month follow-up period may be insufficient to capture late complications such as osteonecrosis, nonunion, or implant failure. In addition, fluoroscopic acquisition frequency was recorded as a procedural measure, but radiation dose, cost, resource utilization, and patient-reported outcomes were not evaluated. Finally, although radiographic measurements were independently performed by two trained orthopaedic surgeons and repeated by one assessor after a 2-week interval, formal quantitative assessment of interobserver and intraobserver reliability was not available for this retrospective dataset. Therefore, measurement variability should be considered when interpreting relatively small differences in radiographic parameters. Furthermore, femoral-neck shortening was assessed using the contralateral hip as an internal reference rather than a separately validated standardized measurement protocol.

In conclusion, robot-assisted percutaneous cannulated-screw fixation was associated with greater procedural efficiency, fewer fluoroscopic acquisitions, and more favourable screw-placement parameters compared with conventional freehand fixation in this cohort. Statistically significant differences were also observed in postoperative day-1 pain, HHS values, and femoral-neck shortening; however, the absolute differences in HHS and femoral-neck shortening were small, and their clinical relevance remains uncertain. Radiation dose was not directly measured, and fluoroscopic acquisition frequency should not be interpreted as a direct measure of radiation exposure. Operative performance showed a decreasing trend across the observed sequence of robot-assisted cases; however, the limited sample size and exploratory nature of the analysis precluded establishment of a validated proficiency threshold.

## Data Availability

The original contributions presented in the study are included in the article/Supplementary Material, further inquiries can be directed to the corresponding authors.
